# Circadian Gating of Thyroid Hormone Action in Hepatocytes

**DOI:** 10.3390/cells13121038

**Published:** 2024-06-14

**Authors:** Karla Lincoln, Jingxuan Zhou, Henrik Oster, Leonardo Vinicius Monteiro de Assis

**Affiliations:** 1 Institute of Neurobiology, Center of Brain Behavior & Metabolism, University of Lübeck, 23562 Lübeck, Germany; kll@nexs.ku.dk (K.L.); jingxuan.zhou@student.uni-luebeck.de (J.Z.); 2 University Hospital Schleswig-Holstein, Campus Lübeck, 23538 Lübeck, Germany

**Keywords:** circadian clock, thyroid hormones, lipid metabolism, endocrinology, fatty liver

## Abstract

Thyroid hormones, thyroxin (T_4_) and the biologically active triiodothyronine (T_3_), play important roles in liver metabolic regulation, including fatty acid biosynthesis, beta-oxidation, and cholesterol homeostasis. These functions position TH signaling as a potential target for the treatment of metabolic dysfunction-associated steatotic liver disease (MASLD). Elevated T_3_ levels in the circulation are associated with increased hepatic lipid turnover, which is also under the control of the circadian clock system. In this study, we developed a cell system to study the impact of hepatocyte circadian rhythms on the metabolic response to T_3_ treatment under control and steatotic conditions. Synchronized AML-12 circadian reporter hepatocytes were treated with T_3_ at different circadian phases and metabolic conditions. T_3_ treatment increased metabolic activity in a dose-independent fashion and had no significant effect on circadian rhythms in AML-12 cells. T_3_ had marked time-of-treatment-dependent effects on metabolic transcript expression. Steatosis induction altered metabolic transcript expression in AML-12 cells. In this condition, the circadian rhythm period was lengthened, and this effect was independent of T_3_. Under steatotic conditions, T_3_ had marked time-of-treatment dependent effects on metabolic transcript expression, which differed from those observed under control conditions. These findings reveal a time-of-day-dependent response of hepatocytes to T_3_, which is further modulated by the metabolic state. Our data suggest that time has a strong influence on liver TH action, which might be considered when treating MASLD.

## 1. Introduction

Thyroid hormones (THs) are systemic regulators of energy metabolism that influence several cellular activities, such as oxygen consumption and energy expenditure. THs enhance ATP production and modulate the metabolism of carbohydrates and lipids [1]. Regulation of TH biosynthesis occurs through the hypothalamic–pituitary–thyroid axis, where the thyrotropin-releasing hormone from the paraventricular nucleus prompts the anterior pituitary to release thyroid-stimulating hormone (TSH). In response to TSH, the thyroid gland secretes thyroxine (T_4_) and tri-iodothyronine (T_3_), which exert negative feedback on the pituitary and hypothalamus [1,2]. Cellular uptake of THs involves transporters such as monocarboxylate transporter 8 (MCT8) and MCT10 [3]. Inside target cells, T_4_ is converted to the biologically active T_3_ by deiodinase enzymes (DIOs) [4]. T_3_ binding to TH receptors (THRA/B) initiates gene transcription by interacting with thyroid hormone response elements [1]. 

THRB is the most abundant TH receptor in the liver, mostly present in hepatocytes, while THRA is predominantly expressed in non-hepatocytes, such as resident macrophages [5,6]. TH signaling predominantly increases hepatic lipid beta-oxidation and cholesterol clearance as bile acids. The importance of TH signaling in the liver is seen when patients show either high or low TH levels, leading to an increase or decrease in lipid and cholesterol metabolism [5,7]. Metabolic dysfunction-associated steatotic liver disease (MASLD), characterized by excessive hepatic lipid accumulation (i.e., steatosis), shows a concerning rise in prevalence worldwide [8]. Hepatic fat buildup can advance to metabolic dysfunction-associated steatohepatitis, which is marked by inflammation and tissue damage [9]. Subsequent complications include endoplasmic reticulum stress, systemic inflammation, insulin resistance, altered gut microbiota, and disrupted circadian control, culminating in a complex disease phenotype [9,10,11]. Given that THs lead to an overall reduction of hepatic lipid and cholesterol levels, the therapeutic application of TH analogs has been proposed and is currently being explored in clinical trials [12,13,14]. 

Physiological adaptation to daily environmental cycles is governed by the circadian clock, an endogenous timing system that aligns internal processes with the 24 h day through transcriptional–translational feedback loops. The liver is an important metabolic hub involved in energy metabolism (e.g., carbohydrate, lipid, cholesterol, and xenobiotic metabolism) that is subject to a regulation exerted by the circadian system [11,15] and THs [5]. While serum TH levels exhibit only low-amplitude circadian rhythms [16,17,18,19], many established TH target genes show marked daily rhythms in their expression. Moreover, altering systemic TH levels has been shown to affect liver metabolic transcriptome rhythms in terms of mesor (average expression level), phase (e.g., time of peak expression), and amplitude (i.e., variation across the day), while having minimal effects on the expression of the hepatic clock gene machinery itself [19,20]. Although TH serum levels show shallow circadian regulation, this suggests that the liver can display periods of low and high sensitivity to TH stimulation in a time-of-day-dependent manner.

To study a possible interaction of time in regulating TH action in liver metabolism, we designed an in vitro model to study the temporal effects of T_3_ on hepatocytes under physiological and steatotic conditions. We selected genes with a prominent role in metabolic regulation as transcriptional factors (*Foxo1*, *Hnf4a*, *Mlxipl*/*Chrebp*) and energy metabolism (*Lpl*, *Plin2*, *Nampt*) and known to respond to alteration in TH levels. We observed a significant time-of-day-dependent response to T_3_ in the expression of metabolic genes in non-steatotic conditions. This time-of-day dependency was altered under steatotic conditions. Illustrating this example, *Hnf4a* was upregulated when T_3_ was given 8 h after synchronization, while this effect was lost under a steatosis condition. This suggests a circadian gating mechanism (e.g., a process in which a treatment may generate a different effect when presented at different times of the day) of TH action that also depends on the metabolic state. 

## 2. Material and Methods

### 2.1. Cell Culture Maintenance

An immortalized liver cell line (AML-12), obtained from the American Type Culture Collection Biobank (CRL-2254) was kept in Gibco Dulbecco’s Modified Eagle Medium with Nutrient Mixture F12 (DMEM/F12, ThermoFisher, Waltham, MA, USA) that included 1% penicillin-streptomycin (ThermoFisher), 1% insulin-transferrin-selenium (ITS, ThermoFisher), 10% non-heat-inactivated fetal bovine serum (FBS, ThermoFisher), and 10 nM of water-soluble dexamethasone (Sigma-Aldrich, St. Louis, MO, USA) in a humidified incubator with 5% CO_2_ at 37 °C. The media composition was kept unchanged for all experiments, except when dexamethasone was omitted, and regular FBS was replaced with 10% charcoal-treated FBS (stripped FBS, Hyclone, Logan, UT, USA) as required to reduce the levels of endogenous hormones, such as T4 (by 40%) and T_3_ (by 89%). Stripped FBS was chosen to diminish the presence of endogenous hormones.

The AML-12 *Bmal1:Luc*, which harbors the luciferase gene under the control of the *Bmal1* promoter, was generated by transducing cells with lentivirus. HEK 293T cells with 70–80% confluence were transfected using a calcium chloride (CaCl_2_) solution. A CaCl_2_ solution containing 17.5 μg of the *Bmal1:Luc* plasmid (pABpuro-Bluf, [21]) and the packaging plasmids psPax2 and pMD2G (12.5 and 7.5 μg, respectively) was prepared and added to the cells. Forty-eight hours post-transfection, the supernatant containing the lentivirus was collected. The virus was then concentrated 10-fold using Lenti-X Concentrator columns (Takara Bio, Kusatsu, Japan) as per the manufacturer’s instructions. The transduction of AML-12 wild-type cells was carried out, followed by selection with 3 μg/mL of puromycin. Single clones were isolated and expanded. Clone 4 of AML-12 *Bmal1:Luc* was selected for subsequent assays. Cells were routinely checked for mycoplasma contamination.

### 2.2. Cellular Metabolism

Five thousand cells were seeded in 96-well plate and were allowed to attach for twenty hours. Increasing levels of T_3_ (1 to 100 nM) in stripped media were given and remained for 48 h. Then, the media were removed, and an XTT solution containing an electron coupling reagent (7:1) was added and measured after 8 h. T_3_ concentration was based on previous studies [22,23,24]. XTT (CyQUANT™ XTT, ThermoFisher) specific absorbance was calculated as [Abs450 nm(Test) − Abs450 nm(Blank)] − Abs660 nm(Test), as previously described [25].

### 2.3. Bioluminescence Recording

In 35-mm Petri dishes, 2 × 10^5^ cells were seeded for bioluminescence recording. The cells were then incubated for 24 h. Cells were synchronized with dexamethasone (200 nM) for two hours. Cells were then divided into four groups: (1) control treated with bovine serum albumin (BSA) at 0.041 mM; (2) control treated with BSA (0.041 mM) and T_3_ (100 nM); (3) palmitate–BSA solution (6:1 ratio, 0.25 mM, Cayman, Ann Arbor, MI, USA); (4) palmitate (0.25 mM) with T_3_ (100 nM). After synchronization, the cells were treated by gently aspirating the old media and adding fresh media containing 200 μM of luciferin (AppliChem GmbH, Darmstadt, Germany) along with the treatments. Following the addition of treatment, the dishes were sealed with round glass coverslips and parafilm. Bioluminescence signals were then recorded at 10 min intervals at 37 °C with continuous measurement.

### 2.4. Gene Expression Evaluation

In total, 10^5^ cells were seeded into 12-well plates and were allowed to attach for 24 h. Cells were synchronized with dexamethasone (200 nM) for two hours. Cells were then divided into four groups: (1) bovine serum albumin (BSA) at 0.041 mM; (2) BSA (0.041 mM) with T_3_ (100 nM); (3) palmitate–BSA solution (6:1 ratio, 0.25 mM, Cayman, Cayman, Ann Arbor, MI, USA); (4) combined palmitate (0.25 mM) with T_3_ (100 nM). After synchronization, media were removed by aspiration and fresh media with the respective treatments were given 8 and 20 h after synchronization. Cells were harvested 24 h after each treatment.

### 2.5. Oil Red O (ORO)

Cells were seeded as described for qPCR experiments. Cells were synchronized with dexamethasone (200 nM) for two hours. Cells were then divided into four groups, as described above. After 48 h, cells were fixed with Cytofix/Cytoperm™ (BD Biosciences, Franklin Lakes, NJ, USA) for 30 min at room temperature, washed with PBS, and stored at 4 °C in PBS-sealed plates. For ORO staining, a 3 mg/mL solution from a 5 mg/mL isopropanol stock (Sigma-Aldrich) was prepared, filtered, and applied to the cells for 2 min after washing three times with ultrapure water. Post-staining, cells were washed three times with water. Staining was quantified with a Nikon Eclipse Ts2R microscope at 20× magnification, capturing four–six images per replicate (six replicates per group) and averaging them. ImageJ software (version number 1.54g) converted images to green channel, with a threshold of 188 to identify and quantify red-stained lipid droplets. This threshold was uniformly applied to all images, and the percentage of red-stained area was calculated for analysis.

### 2.6. RNA Isolation and qPCR

Total RNA was extracted using Trizol reagent (ThermoFisher, Waltham, MA, USA) as per the manufacturer’s protocol, incorporating 1-bromo-3-chloropropane (Sigma-Aldrich, St. Louis, MO, USA) for phase separation, followed by isopropanol precipitation and 75% ethanol washes to purify the RNA. RNA purity was confirmed by obtaining 280/260 and 260/230 absorbance ratios greater than 1.8 using a spectrophotometer. Up to 2 μg of the total RNA was reverse transcribed with random hexamer primers using the RevertAid First Strand cDNA Synthesis Kit (Thermo Fisher Scientific). qPCR was performed using the Go Taq qPCR Master Mix (Promega, Madison, WI, USA) using 50 ng of cDNA. The following amplification program was used on a Bio-Rad CFX96 cycler (Bio-Rad, Hercules, CA, USA): 5 min at 94 °C, 45 cycles of 15 at 94 °C, 15 s at 60 °C, and 20 s at 72 °C, and final extension for 5 min at 72 °C. After amplification, a melt curve was generated to verify product specificity by heating the product from 65 °C to 95 °C at 0.5 °C/s. Primer sequences are provided in Table 1. Relative expression ratios for each transcript were calculated based on individual primer efficiencies using the Pfaffl method [26]. Fold change was calculated compared to the BSA group (unless otherwise specified) for each time point and compared. *Eef1a1* was used as the reference gene that showed robust expression across time points and conditions (two-way ANOVA, effect of time, *p* = 0.65, effect of treatment, *p* = 0.7).

## 3. Results

### 3.1. AML-12 Cells Are a Suitable Model to Study TH–Clock Interaction

**Figure 1:**
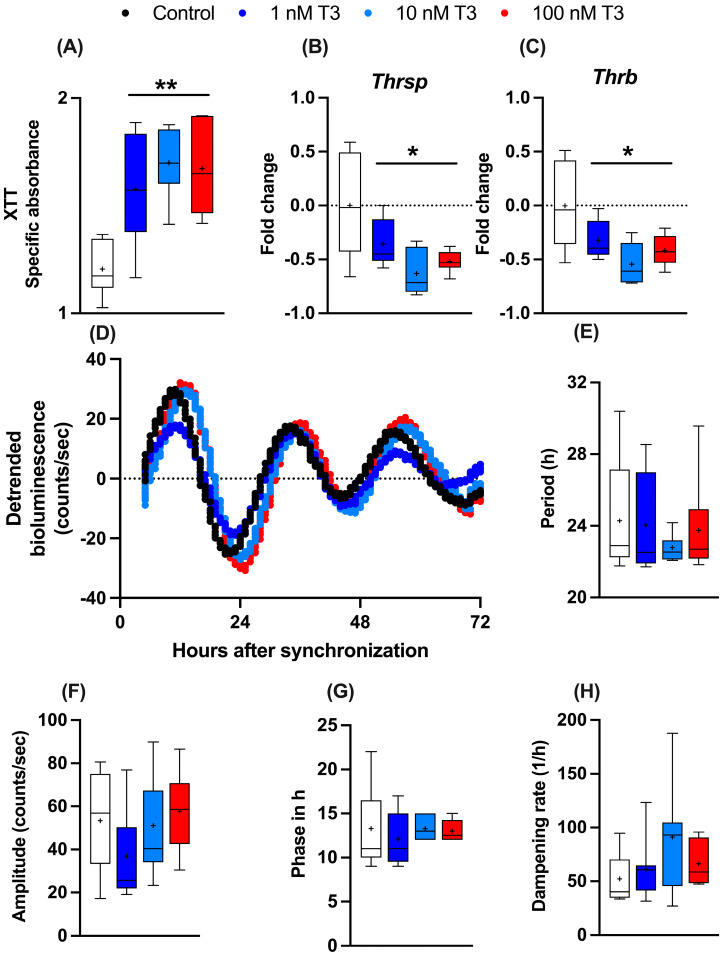
Effects of T_3_ on hepatocyte metabolism and gene regulation. (**A**–**C**) Analysis of metabolic activity using XTT assay and expression levels of thyroid hormone-responsive genes (non-log transformed), *Thrsp* and *Thrb* (n = 6 per group). (**D**–**H**) Evaluation of *Bmal1:Luc* bioluminescence rhythm parameters in response to increasing concentration of T_3_ (n = 6–10 samples per group). Boxplots show the median, mean (+), quartiles, maximum, and minimum expression values. * Represents one-way ANOVA followed by two-stage linear step-up procedure of Benjamini, Krieger, and Yekutieli post-test against the control group. * *q* < 0.05 and ** *q* < 0.01.

To study the influence of T_3_ on liver metabolism, we manufactured an immortalized mouse hepatocyte cell line (AML-12) stably expressing the circadian luciferase reporter *Bmal1:luc*. AML-12 cells were kept in “stripped” media containing reduced amounts of endogenous hormones, such as T_3_ and T_4_. To evaluate the general effects of T_3_ on cell metabolism, 24 h after seeding, cells received increasing levels of T_3_ (1 to 100 nM), and 48 h later, cellular metabolic activity (e.g., mitochondrial dehydrogenase activity) was measured by XTT assay. A dose-independent increase in metabolic activity was observed (Figure 1A). Gene expression analysis of two important TH regulator genes, thyroid hormone responsive (*Thrsp*) and thyroid hormone receptor B (*Thrb*), were downregulated after T_3_ treatment in comparison to the untreated control (Figure 1B,C). To investigate the effects of T_3_ on hepatocyte circadian rhythms, cells were synchronized with dexamethasone and subjected to real-time luminescence recording. Synchronization yielded robust luminescence rhythms for at least 3–4 days (Figure 1D) independent of T_3_ conditions. Moreover, T_3_ treatment did not significantly affect *Bmal1:luc* rhythm parameters, such as period (the length of one complete cycle), amplitude, acrophase, and dampening rate (Figure 1E–H). This suggests that T_3_ treatment has little impact on the regulation of the circadian clock of AML-12 cells. 

### 3.2. The Sensitivity of Hepatocytes to T_3_ Action Is Time-of-Day-Dependent

**Figure 2:**
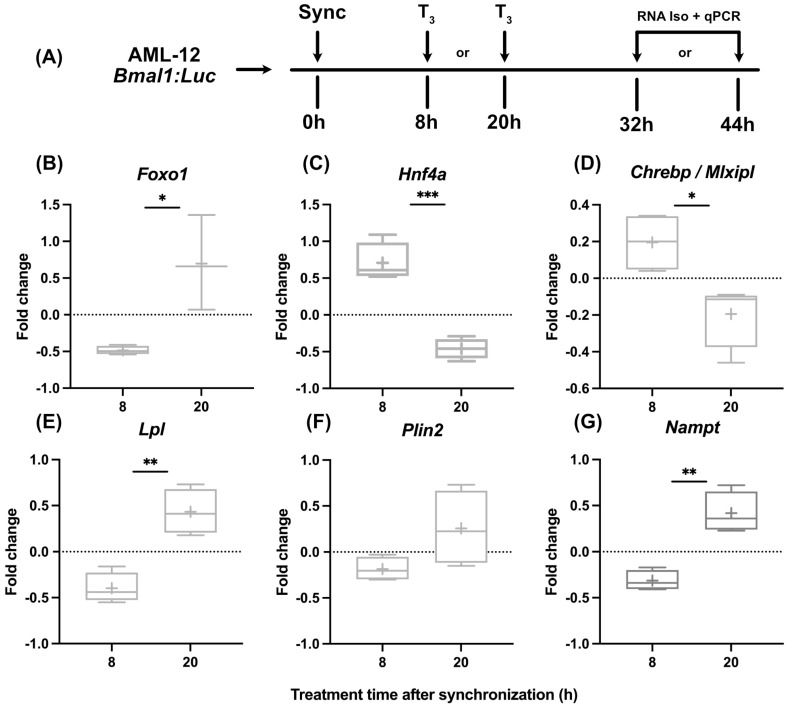
Temporal dynamics of gene expression in response to T_3_ treatment. (**A**) Illustration detailing the experimental design to investigate the effect of T_3_ on hepatocytes under control conditions. (**B**–**G**) Sequential depiction of alterations in gene expression following administration of 100 nM T_3_ at two time points. Fold change (non-log transformed) is shown against the BSA group (n = 3–4). Boxplots show the median, mean (+), quartiles, maximum, and minimum expression values. Comparison between time point 8 h and 20 h in T_3_-treated groups was performed using an unpaired Student’s *t*-test. Significance levels are as follows: * *p* < 0.05, ** *p* < 0.01, *** *p* < 0.001.

While in line with published findings [19] that indicated subtle effects of T_3_ on liver clock function, we tested if hepatocytes show different metabolic responses to T_3_ treatment when treated at different times of the day. After dexamethasone synchronization, hepatocytes were treated with T_3_ either after 8 h or 20 h, corresponding roughly to early morning and evening conditions in vivo, respectively. RNA was harvested 24 h later and analyzed (Figure 2A). Although we found a dose-independent effect for T_3_ on mitochondrial activity, we decided to use the highest dose of T_3_ (100 nM) since the incubation period of the following experiments was reduced to 24 h. We selected three genes encoding for metabolically relevant transcription factors (*Foxo1*, *Hnf4a*, and *Chrebp*) and three further metabolic regulator genes (*Lpl*, *Plin2*, and *Nampt*) reported to be hepatic TH target genes and/or identified as altered under low and/or high TH conditions [19,27,28,29]. 

Our observations revealed marked temporal differences in transcriptional responses to T_3_. Regulation of *Foxo1*, *Lpl*, and *Nampt* was more positive when cells were treated at 20 h, whereas the opposite response was observed for *Hnf4a* and *Chrebp* expression, which was inhibited in response to T_3_ at 20 h (Figure 2B–G). 

### 3.3. AML-12 Cells Are a Suitable Model to Study TH–Clock Interaction in Steatosis

**Figure 3:**
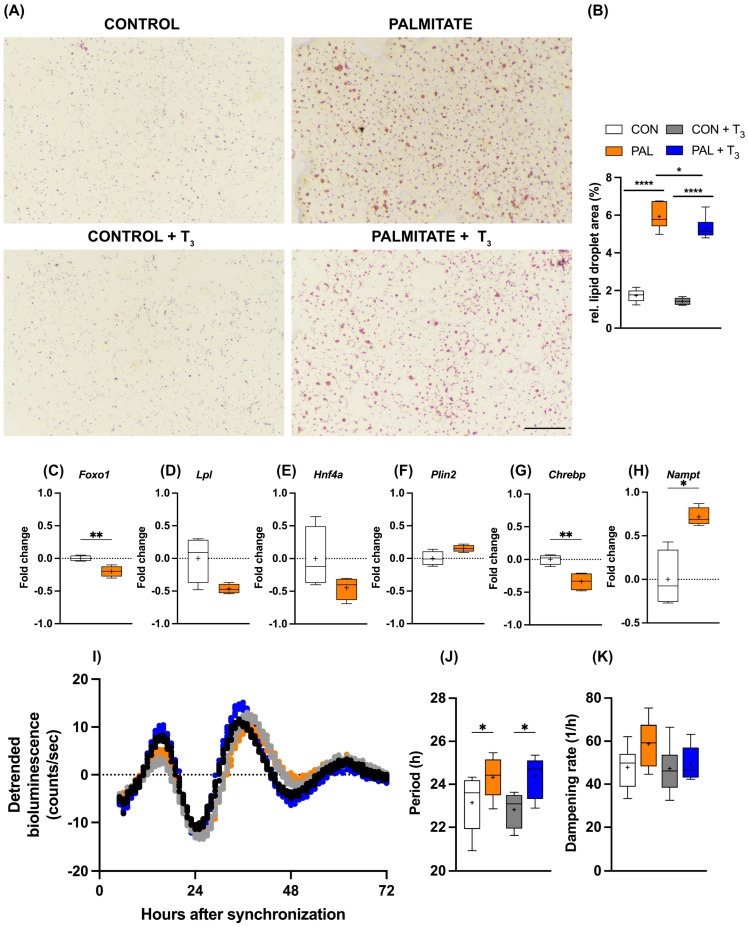
Impact of T_3_ on hepatic steatosis and circadian rhythms. (**A**,**B**) Visualization and quantification of lipid accumulation using oil red O (ORO) staining following treatment with 100 nM T_3_ in cells treated with palmitate. Scale bar represents 100 μm (n = 5–6 per group). In (**B**), * represents two-way ANOVA followed by two-stage linear step-up procedure of Benjamini, Krieger, and Yekutieli test against the control group. * *q* < 0.05, **** *q* < 0.0001. (**C**–**H**) Quantitative PCR analysis depicting gene expression (non-log transformed) profiles under control (BSA-treated) and palmitate-loaded conditions, 20 h after synchronization. Unpaired *t*-test was performed, and significance is denoted as * *p* < 0.05 and ** *p* < 0.01. (**I**–**K**) Representative *Bmal1:luc* bioluminescence tracers and rhythm parameters quantification (n = 7–8). * represents two-way ANOVA followed by two-stage linear step-up procedure of Benjamini, Krieger, and Yekutieli post-test against the control group (* *q* < 0.05). Boxplots show the median, mean (+), quartiles, maximum, and minimum expression values.

As outlined above, TH signaling is a candidate target for the treatment of MASLD in human patients. Against this background, we wondered whether the observed temporal gating of T_3_ action in AML-12 cells would persist under steatosis conditions. The addition of palmitate to the culture medium increased cellular lipid deposition in AML-12 cells, and simultaneous T_3_ administration attenuated this effect (Figure 3A,B). Induction of steatosis also affected metabolic gene expression. *Foxo1* and *Chrebp* mRNA levels were significantly reduced under palmitate, while a similar trend was observed for *Lpl* and *Hnf4a* (*p* < 0.1). In contrast, *Nampt* showed increased expression in response to palmitate treatment (Figure 3C–H). Palmitate incubation had a slight lengthening effect on the period of the circadian clock of AML-12 cells, which was not further affected by T_3_ (Figure 3I–K).

### 3.4. Modulation of the Circadian Gating of T_3_ Action under Steatotic Conditions

**Figure 4:**
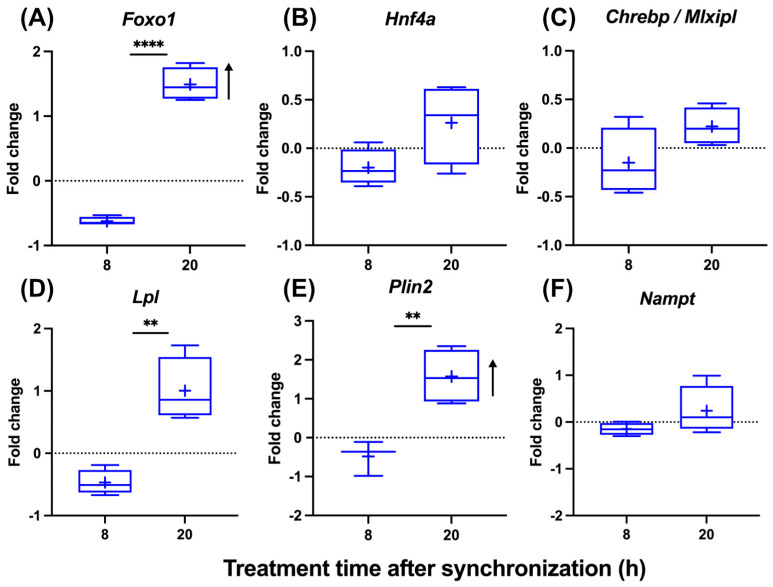
Temporal analysis of T_3_ treatment effects on gene expression in hepatocytes under steatosis. (**A**–**F**) Graphical representation of gene expression changes in response to 100 nM T_3_ treatment across different times of the day in cell treatment with palmitate (n = 3–4 per group). Boxplots show the median, mean (+), quartiles, maximum, and minimum expression values. Fold change (non-log transformed) is shown against the palmitate group. Comparison between time point 8 h and 20 h in T_3_-treated groups was performed using an unpaired Student’s *t*-test. Significance levels are as follows: ** *p* < 0.01, **** *p* < 0.0001. The arrow’s direction represents an increased or decreased expression between BSA and palmitate groups treated with T_3_ using two-way ANOVA followed by two-stage linear step-up procedure of Benjamini, Krieger, and Yekutieli post-test (*q* < 0.05).

To analyze the impact of steatosis on the circadian gating of T_3_ action, we treated steatotic AML-12 with T_3_ at two different times after synchronization. A time-of-day-dependent response to T_3_ was conserved under steatotic conditions, albeit with modifications. *Foxo1* induction was stronger at 20 h compared to 8 h after synchronization. Importantly, *Foxo1* induction only at 20 h was also increased compared to control (non-steatotic) conditions (Figure 4A and Figure 2B). *Hnf4a* and *Chrebp* showed a slight downregulation at 8 h (*p* < 0.1), while mRNA levels were largely unresponsive at 20 h (Figure 4B,C). Temporal responses in *Lpl*, *Plin2*, and *Nampt* expression were more comparable between control and steatotic conditions, with more positive responses at 20 h, except for *Nampt* under steatosis conditions (Figure 4D–F). The induction of *Foxo1* and *Plin2* was significantly stronger under steatosis compared to control conditions 20 h after synchronization (Figure 4A–F and Figure 2B–G).

Taken altogether, these data show that response patterns of AML-12 hepatocytes to T_3_ depend on time and metabolic state. Combined with a largely unresponsive clock system, these data suggest an interactive impact of hepatocyte clocks and metabolic state on hepatic TH action.

## 4. Discussion

In this study, we describe a temporal and state-dependent gating of hepatocyte responses to T_3_ treatment. Under steatotic conditions, T_3_ gating was altered with more pronounced responses during the late phases of the circadian cycle. Importantly, these temporal alterations were not associated with marked changes in the hepatic circadian clock machinery.

To study the circadian T_3_ gating, we used synchronized hepatocytes treated at different times of the day and under different metabolic conditions. We focused on transcriptional TH effects by selecting genes (transcriptional factors and metabolic genes) known to respond to changes in TH levels and to play an important role in liver energy metabolism. Transcriptional factors—such as FOXO1 (forkhead box O1), known to affect glucose and lipid metabolism [30], HNF4A (hepatocyte nuclear factor 4 alpha), a master transcriptional regulator of liver glucose and lipid metabolism [31], and MLX interacting protein-like (MLXIPL or CHREBP), a glucose sensor known to regulate glycolysis and lipogenesis [32]—were selected. Metabolic genes—such as Lipoprotein lipase (LPL) that cleave triglycerides into free fatty acids [33], perilipin 2 (PLIN2), whose expression is associated with lipid storage under steatotic conditions [34], and nicotinamide phosphoribosyltransferase (NAMPT) that catalyzes a key step in the biosynthesis of nicotinamide dinucleotide (NAD+) [35]—were also selected. Importantly, all these genes respond directly to T_3_ treatment [19,27,28,29].

It has been shown that either low or high TH levels have only a slight phase effect on core clock transcriptional rhythms, although marked alterations on the rhythmic transcriptome, especially at high TH level conditions, are found [19,20]. Importantly, serum levels of T_3_ or T_4_ are mostly arrhythmic in contrast to highly rhythmic hormones, such as cortisol [16,17,18,19]. Since THs are not strongly rhythmic, one question that arises is how a hormone class that carries virtually no temporal information can lead to a temporal response. One can suggest that, although THs levels are constant, its effects can be time-of-day-controlled by a temporal control in TH modulators, i.e., transporters, DIOs, and/or receptor binding. In the liver, THs transporters are not strongly regulated in either amplitude or phase, being mostly affected at the mesor level by the TH level. Hepatic *Dio1* expression shows no rhythmic pattern but responds to the TH level in a T_3_-dose-dependent manner. At the receptor level, *Thra*, albeit rhythmic, shows a slight phase advance only in a low TH condition, while *Thrb* is arrhythmic. Importantly, both receptors respond with mesor changes in response to TH levels [19,20].

Circadian gating in physiological systems refers to a mechanism that regulates the responsiveness to specific temporal cues in a time-of-day-dependent manner. This mechanism ensures that responses occur only at appropriate times to maintain alignment with rhythms in environmental demands. Considering the modest rhythmic regulation of TH modulators (i.e., transporters, deiodinases, and receptors) at the transcriptional level [19,20], such a gating mechanism may also occur independently of rhythmic TH modulation. Another possible mechanism of gating of TH action could be associated with the ability of THRs to bind to DNA regions and affect transcriptional output across the day. Finally, non-canonical TH signaling could represent a possible route for circadian gating, albeit the slow kinetics of T_3_ action in our experimental setup are more reminiscent of canonical TH (transcription-based) action. Importantly, our findings suggest that circadian gating is impacted by steatosis, which adds another layer of regulation, the cellular context. Further experimentation addressing the points described above using different knockout models is required to validate these hypotheses.

It is known that dexamethasone treatment leads to a resetting of clock gene expression rhythms and, hence, overall clock phase [36]. Considering that *Bmal1* mRNA acrophase in vivo is in the late night/early morning (ZT 23–24) and that *Bmal1:luc* in our conditions peaked 12 h after dexamethasone treatment, treatment at 8 h or 20 h after synchronization roughly represents subjective day or night conditions, respectively. In this regard, under non-steatotic (control) conditions, T_3_ effects on target gene expression were stronger at 20 h (night treatment) after synchronization, while less activation or even downregulation was observed after treatment at 8 h (morning treatment), suggesting a time-of-day-dependent effect on T_3_ sensibility in hepatocytes. Notably, the effects of T_3_ were potentiated under steatotic conditions—mostly in the subjective night—for *Foxo1* and *Plin2*. One must consider that the higher effects of T_3_ under steatosis may be attributed to a different baseline compared to a non-steatotic condition.

Our findings might have some clinical relevance, especially for MASLD. One could suggest that TH/THR-targeted MASLD treatment, e.g., via THRB agonists such as resmetiron, could be improved if the administration time is adjusted based on clock function and disease state, especially due to its short half-life (<5 h) [37]. Interestingly, hepatocytes showed increased sensitivity to T_3_ during the subjective night (the active phase in mice). In vivo data support this finding, showing that the highest catabolism of lipids and cholesterol takes place during the active phase and is strongly enhanced by T_3_ [19]. Therefore, chrono-modulated regimes could improve therapeutic outcomes considering the recent approval of resmetiron by the U.S. Food and Drug Administration (FDA).

To our knowledge, no study has evaluated how T_3_ effects given at different times of the day affect the liver transcriptional responses. In addition, our experimental setting contains a synchronization step (e.g., dexamethasone), which is often restricted to circadian studies. Therefore, comparison to previous single-time point studies from the literature is challenging and limited. Notably, our study has some limitations. We used an immortalized cellular model that inherently has a different phenotypic response from primary hepatocytes. Our study lacks a mechanistic view of the gating mechanism. One future experiment is to evaluate the contribution of the hepatocyte clock in the gating response, which would clarify the dependency of the circadian clock on the gating mechanism. Moreover, our in vitro conditions are devoid of systemic factors, such as immune system and adipose tissue-derived factors, which are factors that may regulate the gating mechanism in vivo.

## 5. Conclusions

Taken altogether, our in vitro data suggest that T_3_ effects are time-of-day-dependent and further modulated by steatosis. Our experimental model is an interesting tool for exploring the circadian aspect of TH signaling at different levels of regulation, i.e., transcriptional, post-transcriptional, and post-translation levels. We suggest that the time of T_3_ treatment has a strong effect on liver transcriptional output and should be carefully selected in in vitro and possibly in in vivo studies. The temporal effects of T_3_ identified in this study may implicate the treatment of MASLD and lead to the proposal of chrono-modulated regimes in MASLD.

## 6. Statistical Analysis

Bioluminescence data were analyzed using Lumicycle Analysis software (version number 3.1), where curves were calculated by employing the 24 h running average method. A dampened sine curve was fitted to the data using the least squares regression method in GraphPad Prism software. Rhythmic parameters, such as amplitude, acrophase (in hours), period (in hours), and dampening (half-life in hours), were calculated for each curve. Constraints were set for the period between 18 and 20 h and kept consistent for all groups. Values exceeding the mean ± 2 standard deviations were deemed outliers and excluded from the analysis. A one-way ANOVA was used for comparisons across conditions, while a two-Way ANOVA was employed for comparisons across both time and conditions. In both cases, the post-test followed a two-stage linear step-up procedure of Benjamini, Krieger, and Yekutieli, with *q* values < 0.05. For temporal comparisons within the sample group or between two conditions, an unpaired Student’s *t*-test was used and a *p*-value < 0.05 was set as significant. Graphs and statistical analyses were performed using GraphPad Prism software (version number 10.2).

## Figures and Tables

**Table 1:** Primer sequences.

Gene Name	Access Number	Sequence Forward (5’-3’)	Sequence Reverse (5’-3’)	Efficiency (%)
*Chrebp*	NM_021455.5	CCTGCATCGATCACAGGTCA	AGACCAGCTTGCCACTGTAAG	86
*Foxo1*	NM_019739.3	GGCGGGCTGGAAGAATTCAA	CTCTTGCCTCCCTCTGGATTG	104
*Hnf4a*	NM_008261.3	TGACCATGGGCAATGACACG	TGTGGTTCTTCCTCACGCTC	107
*Lpl*	NM_008509.2	TTGCCCTAAGGACCCCTGAA	ACATTCCCGTTACCGTCCATC	108
*Nampt*	NM_021524.2	GAACAGATACTGTGGCGGGAA	CAAGCCGTTATGGTACTGTGCT	105
*Plin2*	NM_007408.4	ACTCCACTGTCCACCTGATTG	GATGTGCTCAACACAGTGGG	97
*Thrb*	NM_001113417.1	CCTGGATCCTGACGATGTGAA	CTTCTAAAGAAGCCCTTGCAGC	65
*Thrsp*	NM_009381.3	CAGGAGGTGACGCGGAAATA	TAAAGGTGAGCCTGCAACCA	90
*Eef1a1*	NM_010106.2	TGCCCCAGGACACAGAGACTTCA	AATTCACCAACACCAGCAGCAA	94

## Data Availability

All data is available upon request to the corresponding author.
